# Associations of Visceral Adipose Tissue and Skeletal Muscle Density With Incident Stroke, Myocardial Infarction, and All‐Cause Mortality in Community‐Dwelling 70‐Year‐Old Individuals: A Prospective Cohort Study

**DOI:** 10.1161/JAHA.120.020065

**Published:** 2021-04-17

**Authors:** Marcel Ballin, Peter Nordström, Johan Niklasson, Anna Nordström

**Affiliations:** ^1^ Unit of Geriatric Medicine Department of Community Medicine and Rehabilitation Umeå University Umeå Sweden; ^2^ Section of Sustainable Health Department of Public Health and Clinical Medicine Umeå University Umeå Sweden; ^3^ School of Sport Sciences UiT The Arctic University of Norway Tromsø Norway

**Keywords:** body composition, cardiovascular disease, ectopic fat, obesity, Aging, Cardiovascular Disease, Epidemiology, Obesity, Risk Factors

## Abstract

**Background:**

Aging leads to increased visceral adipose tissue (VAT) and reduced skeletal muscle density. To which extent these are associated with the risk of stroke, myocardial infarction (MI), and all‐cause mortality in older adults is unknown.

**Methods and Results:**

A total of 3294 70‐year‐old individuals (49.6% women) underwent a health examination in Umeå, Sweden, during 2012 to 2018. VAT and muscle density were measured using dual‐energy x‐ray absorptiometry and peripheral quantitative computed tomography. Cases of stroke, MI, and all‐cause mortality were collected through national registers. Cox regressions were used to calculate hazard ratios (HRs) and 95% CIs per SD greater VAT and per SD lower muscle density. During a mean follow‐up of 3.6 years, there were 108 cases of stroke or MI, and 97 deaths. Greater VAT (adjusted HR [aHR], 1.56; 95% CI, 1.09–2.22), but not lower muscle density (aHR, 1.14; 95% CI, 0.97–1.34), was associated with increased risk of stroke or MI. Neither VAT (aHR, 0.95; 95% CI, 0.65–1.41) nor muscle density (aHR, 1.11; 95% CI, 0.92–1.34) was associated with all‐cause mortality. The association of VAT with stroke or MI was only significant in men (aHR, 1.86; 95% CI, 1.19–2.91) but not women (aHR, 0.60; 95% CI, 0.25–1.42) (*P*_interaction_=0.038).

**Conclusions:**

With the limitation of being an observational study, these findings suggest that VAT is an important obesity‐related predictor of cardiovascular risk in 70‐year‐old men, and by implication, that decreasing VAT may potentially reduce their risk of cardiovascular disease.

**Nonstandard Abbreviations and Acronyms**
aHR: adjusted hazard ratio
HAI: Healthy Ageing Initiative
MVPA: moderate‐to‐vigorous physical activity
NPR: National Patient Register
uHR: unadjusted hazard ratio
VAT: visceral adipose tissue

**Clinical Perspective**

**What Is New?**

- In community‐dwelling 70‐year‐old individuals, greater visceral adipose tissue was associated with increased risk of stroke or myocardial infarction but not all‐cause mortality, even after adjusting for lifestyle factors, socioeconomic status, cardiovascular risk factors, previous cardiovascular disease, medications, total fat mass, and skeletal muscle density.
- The association was robust when excluding early cases, participants with short follow‐up, and participants with previous cardiovascular disease, but differed according to sex as it was strong and significant only in men but not in women.
- Skeletal muscle density was not associated with incident stroke, myocardial infarction, or all‐cause mortality.

**What Are the Clinical Implications?**

- Failing to account for regional fat distribution when assessing obesity in 70‐year‐old men could mask a potentially increased risk of cardiovascular disease.
- A plausible strategy for reducing the risk of cardiovascular disease in 70‐year‐old men could be exercise interventions, which have been shown to decrease visceral adipose tissue while preserving muscle mass.

Cardiovascular disease (CVD) is the leading cause of mortality, causing around 18 million deaths annually.^1^ Stroke and coronary artery disease, such as myocardial infarction (MI),^1^ cause most of these deaths and have been reported to increase in conjunction with population aging.^2^ One of the major risk factors for stroke, MI, and premature death is obesity,^3^, ^4^, ^5^ and since 1980 there has been a 2‐fold increase in the prevalence of obesity in >70 countries.^6^ In older people, around 60% are estimated to be overweight and 20% to 30% have obesity.^7^, ^8^, ^9^ Consequently, prevention and treatment of obesity and obesity‐associated morbidities in older people is important to promote healthy aging and prevent healthcare systems from becoming overwhelmed as obesity rates increase and the older population grows.^10^

Although widely acknowledged to cause deleterious health outcomes in the general population, obesity, as measured by the body mass index (BMI), has suggested to be protective in older adults, also known as the "obesity paradox."^11^, ^12^ The nature of this relationship is complex, and there are several confounding factors that may explain why a high BMI compared with a low BMI has in some cases been found protective in older adults.^12^ For example, a low BMI in older people could be indicative of unintentional weight loss and a higher prevalence of comorbidity.^12^ Furthermore, even though BMI tends to decrease with age, aging leads to an increase in ectopic fat, such as visceral adipose tissue (VAT) and skeletal muscle fat infiltration, both of which appear to be highly atherogenic and key in cardiometabolic abnormalities.^13^, ^14^, ^15^ Thus, by using a crude measure of total adiposity, such as the BMI, the heterogeneity of adipose tissue as well as regional fat distribution, such as VAT, is neglected.^14^, ^16^ Therefore, it is pivotal to consider regional fat distribution when studying the impact of adiposity on health outcomes in older adults.

However, the role of regional fat distribution in relation to cardiovascular risk in older adults is not clear. Specifically, a recent review highlighted that the relationship between VAT and CVD is controversial in older adults, mainly because of a lack of studies.^17^ Specifically, merely one study has found VAT to be associated with MI in older women,^18^ and no association has been found in older men.^18^, ^19^ As for muscle fat infiltration, which can be expressed as lower muscle density,^20^ cross‐sectional associations with insulin resistance, metabolic risk factors, and coronary artery calcification have been observed in middle‐aged and older adults, although this may partly be explained by the joint presence of greater VAT.^15^, ^21^, ^22^ In terms of mortality, 2 recent studies found an association between lower muscle density and higher mortality in middle‐aged and older men,^23^, ^24^ although a study of older men and women with a wide age range found no association.^25^ Similarly, studies on VAT and mortality are few, and the findings are contradicting.^26^, ^27^, ^28^, ^29^ Clearly, the role of VAT and muscle density in relation to CVD and mortality in older people is not fully understood. Therefore, in this study, we investigated the independent associations of greater VAT and lower skeletal muscle density with incident stroke, MI, and all‐cause mortality in 3294 community‐dwelling 70‐year‐old individuals.

## METHODS

### Study Design and Population

The data sets analyzed during the current study are not publicly available in accordance with the General Data Protection Regulation, but a deidentified data set may be available from the corresponding author on reasonable request. This prospective study was based on the Healthy Ageing Initiative (HAI), which is a population‐based prevention study conducted at a single research clinic in Umeå, Sweden. HAI aims to identify risk factors for noncommunicable diseases, falls, and fractures among all 70‐year‐old individuals in Umeå, and to promote physical activity and healthy dietary habits in this population.^30^ Participants arrive to the clinic in a fasting state, where a research nurse leads them through a 3‐hour long health examination. Eligibility criteria for participating in HAI are resident of Umeå and exact age of 70 years, with no exclusion criteria. Eligible individuals are identified and invited using population registers. Currently, >4900 individuals have chosen to participate, corresponding to 84% of everyone invited. VAT measurement is a standing feature in HAI, although muscle density was only measured during May 2012 to January 2018. Thus, the present cohort comprised all HAI participants during May 2012 to January 2018 with complete body composition measurements. Both HAI and the present study have been approved by the Regional Ethical Review Board in Umeå (No. 07‐031M with extensions) and were conducted in accordance with the World Medical Association's Declaration of Helsinki. All participants provided written informed consent to partake in the study.

### Exposure Assessment

VAT was measured through a dual‐energy x‐ray absorptiometry (DXA) scan of each participant using a Lunar iDXA device and the CoreScan software (GE Healthcare Lunar, Madison, WI). The CoreScan algorithm quantifies VAT by subtracting subcutaneous fat from total abdominal fat in the android region and has a root‐mean‐square SD of 35 to 65 g.^31^ Total fat mass was obtained for inclusion as a covariate in the analysis. Scans were performed by 1 of 5 research nurses, with the device calibrated each morning before measurements using a phantom.

Midcalf skeletal muscle density was measured using a Stratec XCT‐2000 (Stratec Medizintechnik, Pforzheim, Germany) peripheral quantitative computed tomography device. Although not a direct measure of skeletal muscle fat infiltration, lower muscle density is a reliable proxy for greater muscle fat infiltration.^20^ As described previously,^32^ slice thickness was set at 2.0 mm, with a voxel size of 0.5 mm, and scan site was at 66% of tibial length. Automatic threshold‐based iterative edge detection–guided segmentation was performed using the manufacturer's software, with a density threshold of 280 mg/cm^3^ used to segment muscle from bone (contour mode 1 and peel mode 2), and a threshold of 40 mg/cm^3^ (contour mode 3 and peel mode 1) used to separate muscle from fat. Muscle cross‐sectional area was obtained for inclusion as a covariate in the forthcoming analysis and was obtained by subtraction of bone area from total bone area+muscle cross‐sectional area. Muscle mass was derived by subtracting bone mass from total bone+muscle mass, whereby muscle density was computed by dividing muscle mass by muscle cross‐sectional area. The coefficient of variation for muscle density using Stratec XCT‐2000 is 0.8%.^33^ Calibration followed the same procedure as for the DXA device.

### Ascertainment of Stroke, MI, and All‐Cause Mortality

Participants were followed up for incident cases from baseline in HAI until December 31, 2018. Cases of stroke and MI were collected from the Swedish National Patient Register (NPR) using the *International Classification of Diseases, Tenth Revision* (*ICD‐10*), diagnostic codes I61 to I64 and I21. The NPR records all diagnoses in inpatient care in Sweden since 1987, and all secondary outpatient care since 2001. The positive predictive value for the diagnosis of stroke and MI in the NPR is high (69%–99% positive predictive value for stroke and 86%–100% positive predictive value for MI).^34^, ^35^, ^36^ Mortality data were collected from the Swedish Cause of Death Register.^37^ These registers are maintained by the Swedish National Board of Health and Welfare.

### Assessment of Other Variables

Body height and weight were measured using a stadiometer (Holtain Limited, Crymych, Dyfed, UK) and a digital scale (Avery Berkel HL 120, Taiwan), whereby the BMI (kg/m^2^) was calculated. Blood pressure was measured using the electronic device Omron M6 Comfort HEM‐7221‐E (Omron Healthcare, Kyoto, Japan), after a 15‐minute rest. Fasting blood glucose was measured using the HemoCue 201 RT system (Radiometer Medical ApS, Denmark), with an accuracy (coefficient of variation) of 2.3%, as determined by daily quality control. Blood lipids were collected through venipuncture and analyzed at the accredited laboratory at the Department of Clinical Chemistry, Umeå University Hospital. The coefficient of variation for measurement of blood lipids in this accredited laboratory is 3%. Moderate‐to‐vigorous physical activity (MVPA) was measured during 1 week using hip‐mounted Actigraph GT3X+ accelerometers, as described previously.^38^ Participants reported their smoking status and alcohol consumption. Data on annual disposable income, level of education, and marital status at the age of 65 years were collected from the registers of Statistics Sweden. Data on medical history and prescription medications were collected from the NPR and Prescribed Drug Register. The Prescribed Drug Register is maintained by the National Board of Health and Welfare and includes data on all prescribed drugs dispensed at pharmacies in Sweden since July 2005. All data in the present study were linked together using unique personal identity numbers, which are issued to all residents of Sweden.

### Statistical Analysis

We used Cox proportional hazard regression models to calculate hazard ratios (HRs) and 95% CIs for the associations of VAT (per 1 SD greater) and skeletal muscle density (per 1 SD lower) with (A) the composite end point of incident stroke or MI and (B) all‐cause mortality. Assessment of the proportional hazards assumption using covariate‐by‐time interaction terms showed that the assumption was not violated. Follow‐up time was calculated as number of days from participation in HAI until the first event of stroke or MI or until death, whichever came first. If no event occurred, follow‐up time ended on December 31, 2018.

First, all models were performed unadjusted. The second model was adjusted for sex, smoking (yes/no), alcohol consumption (never/monthly or less/2–4 times a month/2–3 times a week/≥4 times a week), annual disposable income, education (primary/secondary/postsecondary), and marital status (married/never married/widowed/divorced). The third, multivariable‐adjusted model, was additionally adjusted for prescription medications (antihypertensives/anticoagulants/lipid‐lowering agents), CVD history (previous stroke/MI/angina pectoris), MVPA, low‐density lipoprotein cholesterol, fasting blood glucose, systolic blood pressure, and total fat mass. The models for muscle density were adjusted also for muscle cross‐sectional area. Finally, to assess the independent associations of VAT and muscle density, the multivariable‐adjusted model was repeated with VAT and muscle density entered simultaneously. We investigated whether the associations differed by either sex or MVPA by including product terms in the final multivariable‐adjusted models. In the event of a significant interaction, stratified analyses were performed.

Two sensitivity analyses were conducted. First, we aimed to minimize the risk of reverse‐causality bias, as well as selection bias, where low VAT in some participants may be indicative of early onset of morbidity. Therefore, we repeated all Cox models after excluding the following: (1) all participants with follow‐up time <1 year; (2) all participants who experienced a stroke or MI or died within the first year of follow‐up; and (3) all participants with a history of CVD (previous stroke/MI/angina pectoris). Second, we aimed to explore signs of nonlinearity by adding quadratic terms of VAT and muscle density to the multivariable‐adjusted models. When *P*<0.05 for the quadratic term, we continued by analyzing the association according to tertiles.

Finally, a supplementary analysis was conducted where we investigated the association of BMI with the composite outcome of stroke/MI and all‐cause mortality. For both outcomes, a multivariable‐adjusted model was performed in the total cohort as well as after excluding all participants with <1 year of follow‐up and history of CVD. All analyses were performed using SPSS version 26.0 (IBM Corp, Armonk, NY). Statistical significance was determined as *P*<0.05 and HRs with 95% CIs, which did not cross 1.0.

## RESULTS

### Participant Characteristics

During May 2012 to January 2018, there were 3849 participants in HAI, from which 555 had missing DXA data and/or peripheral quantitative computed tomography data and were excluded. Thus, the total study cohort comprised 3294 participants (49.6% women) with a mean age of 70.4 years. Participant characteristics are shown in Table 1. Mean follow‐up time was 3.6 years (range, 0.1–6.6 years).

**Table 1:** Baseline Characteristics of the 3294 70‐Year‐Old Men and Women Who Participated in the HAI During May 2012 to January 2018

Characteristics	Values
Age, y	70.4±0.1
Female sex, n (%)	1633 (49.6)
Anthropometrics	
Height, cm	170±10
Weight, kg	76.2±13.9
BMI, kg/m^2^	26.3±4.0
Body composition	
Total fat mass, kg	27.1±8.5
VAT, g	1491±959
Muscle density, mg/cm^3^	71.7±4.2
Current smoker, n (%)	189 (5.7)
Alcohol consumption, n (%)	
Never	359 (10.9)
≤Once/month	756 (23.0)
2–4 Times/mo	1258 (38.2)
2–3 Times/wk	727 (22.1)
≥4 Times/wk	137 (4.2)
MVPA, min/d	33.1±25.7
Missing data attributable to insufficient wear time, n	136
Cardiovascular risk factors	
Systolic blood pressure, mm Hg	139.2±16.7
Diastolic blood pressure, mm Hg	81.5±8.8
Fasting blood glucose, mmol/L	5.7±1.1
Low‐density lipoprotein cholesterol, mmol/L	3.26±1.07
High‐density lipoprotein cholesterol, mmol/L	1.58±0.49
Total cholesterol, mmol/L	5.44±1.20
Triglycerides, mmol/L	1.33±0.67
Socioeconomic data*	
Disposable income, 1000 Swedish krona	250±174
Education, n (%)*	
Primary	550 (16.7)
Secondary	1336 (40.6)
Postsecondary	1401 (42.6)
Missing data	7
Marital status, n (%)*	
Married	2239 (68.1)
Never married	290 (8.8)
Widowed	185 (5.6)
Divorced	574 (17.5)
Missing data	6
Prescription medications and comorbidities, n (%)^†^	
Angina pectoris	271 (8.2)
Myocardial infarction	145 (4.4)
Stroke	110 (3.3)
Diabetes mellitus	285 (8.7)
Fracture	526 (16.0)
Kidney failure	22 (0.7)
Cancer	608 (16.8)
Antihypertensives	1876 (57.0)
Lipid‐lowering agents	1409 (42.8)
Anticoagulants	1273 (38.6)

Data are presented as mean±SD unless stated otherwise. BMI indicates body mass index; HAI, Healthy Ageing Initiative; MVPA, moderate‐to‐vigorous physical activity; and VAT, visceral adipose tissue.

* Annual income, level of education, and marital status at the age of 65 years.

^†^ Prescription medication dispensed at a pharmacy in 2005 or later.

### Stroke and MI

There were 108 cases of the composite end point of stroke (n=54) or MI (n=54), with an incidence rate of 9.4 per 1000 person‐years. In unadjusted models, there was a significant association between greater VAT and increased risk of stroke or MI (unadjusted HR [uHR], 1.40; 95% CI, 1.19–1.64), and between lower muscle density and stroke or MI (uHR, 1.22; 95% CI, 1.07–1.38). In the final multivariable‐adjusted model, greater VAT was associated with around 60% increased risk of stroke or MI (adjusted HR [aHR], 1.56; 95% CI, 1.14–2.22), but there was no significant association between lower muscle density and stroke or MI (aHR, 1.14; 95% CI, 0.97–1.34) (Table 2). There was also no significant association between BMI and stroke or MI (Table 3).

**Table 2:** HRs for Incident Stroke, MI, and All‐Cause Mortality per SD Greater VAT and per SD Lower Skeletal Muscle Density in the Total Study Cohort

Variable	No. of Participants (No. of Events)	VAT (per SD Greater)	Muscle Density (per SD Lower)
		HR (95% CI)*	HR (95% CI)^†^
Stroke or MI			
Unadjusted	3294 (108)	1.40 (1.19–1.64)	1.22 (1.07–1.38)
Sex, smoking, alcohol consumption, and socioeconomic status	3227 (107)	1.29 (1.07–1.55)	1.19 (1.03–1.37)
MV model A	3064 (102)	1.57 (1.10–2.24)	1.15 (0.98–1.36)
MV model B	3064 (102)	1.56 (1.09–2.22)	1.14 (0.97–1.34)
All‐cause mortality			
Unadjusted	3294 (97)	1.22 (1.02–1.46)	1.16 (0.99–1.36)
Sex, smoking, alcohol consumption, and socioeconomic status	3228 (95)	1.07 (0.87–1.32)	1.11 (0.93–1.32)
MV model A	3065 (89)	0.97 (0.66–1.42)	1.11 (0.92–1.34)
MV model B	3065 (89)	0.95 (0.65–1.41)	1.11 (0.92–1.34)

MV models were adjusted for sex, smoking, alcohol consumption, education, income, marital status, total fat mass, low‐density lipoprotein cholesterol, fasting blood glucose, systolic blood pressure, previous stroke/MI/angina pectoris, prescribed antihypertensives/anticoagulants/lipid‐lowering agents, and moderate‐to‐vigorous physical activity. In addition, MV models for muscle density also included muscle area. In MV model B, VAT and muscle density were entered simultaneously to assess their independent associations. HR indicates hazard ratio; MI, myocardial infarction; MV, multivariable adjusted; and VAT, visceral adipose tissue.

* For 1 SD, VAT=959 g.

^†^ For 1 SD, muscle density=4.2 mg/cm^3^.

**Table 3:** HRs for Incident Stroke, MI, and All‐Cause Mortality per Unit Increase in BMI

Variable	No. of Participants (No. of Events)	HR (95% CI) per Unit Greater BMI
Stroke or MI		
MV model	3064 (102)	1.00 (0.95–1.05)
MV model excluding those with short follow‐up and previous CVD	2547 (65)	0.99 (0.93–1.06)
All‐cause mortality		
MV model	3065 (89)	0.94 (0.89–1.00)
MV model excluding those with short follow‐up and previous CVD	2582 (59)	0.95 (0.88–1.02)

MV models were adjusted for sex, smoking, alcohol consumption, education, income, marital status, low‐density lipoprotein cholesterol, fasting blood glucose, systolic blood pressure, previous stroke/MI/angina pectoris, prescribed antihypertensives/anticoagulants/lipid‐lowering agents, and moderate‐to‐vigorous physical activity. BMI indicates body mass index; CVD, cardiovascular disease; HR, hazard ratio; MI, myocardial infarction; and MV, multivariable adjusted.

Interaction analyses showed a sex‐specific association of VAT with stroke or MI (*P*_interaction_=0.038). Greater VAT was significantly associated with increased risk of stroke or MI in men (aHR, 1.86; 95% CI, 1.19–2.91), but not women (aHR, 0.60; 95% CI, 0.25–1.42). The incidence of stroke or MI was higher in men than in women (72 versus 36 events; *P*<0.001), and men who experienced an event had more than twice the VAT mass compared with women with an event (2.3 versus 0.9 kg; *P*<0.001). The association between muscle density and stroke or MI did not differ between men and women (*P*_interaction_=0.8). Finally, there were no significant interactions between either VAT and MVPA (*P*_interaction_=0.9) or between muscle density and MVPA (*P*_interaction_=0.7).

### All‐Cause Mortality

There were 97 all‐cause deaths (incidence rate, 8.3/1000). In unadjusted models, greater VAT was significantly associated with higher all‐cause mortality (uHR, 1.22; 95% CI, 1.02–1.46). In the final multivariable‐adjusted models, neither VAT (aHR, 0.95; 95% CI, 0.65–1.41) nor muscle density (aHR, 1.11; 95% CI, 0.92–1.34) was associated with all‐cause mortality (Table 2). Neither of the associations differed between men and women or according to MVPA (*P*_interaction_>0.3 for all). There was also no significant association between BMI and all‐cause mortality (Table 3).

### Sensitivity Analyses

In the sensitivity analysis in which early incident cases and participants with short follow‐up time and previous CVD were excluded, the results were generally confirmed (Table 4). The point estimate for the association of VAT with stroke or MI was slightly higher than in the main analysis (aHR, 1.68; 95% CI, 1.08–2.61). As for muscle density, the point estimate was marginally higher, but the association was now considered statistically significant (aHR, 1.19; 95% CI, 1.02–1.40). Moreover, neither VAT nor muscle density was associated with all‐cause mortality.

**Table 4:** HRs for Incident Stroke, MI, and All‐Cause Mortality in the Sensitivity Analysis, in Which Participants With an Early Event, <1 Year of Follow‐Up, and History of CVD Were Excluded

Variable	No. of Participants (No. of Events)	VAT (per SD Greater)	Muscle Density (per SD Lower)
		HR (95% CI)*	HR (95% CI)^†^
Stroke or MI			
Unadjusted	2712 (70)	1.46 (1.19–1.78)	1.25 (1.09–1.43)
MV model A	2547 (65)	1.73 (1.11–2.70)	1.21 (1.03–1.42)
MV model B	2547 (65)	1.68 (1.08–2.61)	1.19 (1.02–1.40)
All‐cause mortality			
Unadjusted	2751 (63)	1.30 (1.04–1.63)	1.03 (0.80–1.32)
MV model A	2582 (59)	1.40 (0.88–2.24)	0.95 (0.72–1.26)
MV model B	2592 (60)	1.42 (0.88–2.27)	0.94 (0.72–1.25)

MV models were adjusted for sex, smoking, alcohol consumption, education, income, marital status, total fat mass, low‐density lipoprotein cholesterol, fasting blood glucose, systolic blood pressure, previous stroke/MI/angina pectoris, prescribed antihypertensives/anticoagulants/lipid‐lowering agents, and moderate‐to‐vigorous physical activity. In addition, MV models for muscle density also included muscle area. In MV model B, VAT and muscle density were entered simultaneously to assess their independent associations. CVD indicates cardiovascular disease; HR, hazard ratio; MI, myocardial infarction; MV, multivariable adjusted; and VAT, visceral adipose tissue.

* For 1 SD, VAT=934 g.

^†^ For 1 SD, muscle density=4.2 mg/cm^3^.

Evaluation of the quadratic exposure terms confirmed the assumed linearity for the associations of VAT with stroke or MI (*P*_nonlinearity_=0.4); muscle density with stroke or MI (*P*_nonlinearity_=0.8); and muscle density with all‐cause mortality (*P*_nonlinearity_=0.1). However, the association of VAT with all‐cause mortality appeared nonlinear (*P*_nonlinearity_=0.004) and was therefore investigated by tertiles of VAT (Table 5). Compared with tertile 1, the uHR in tertile 2 was 0.80 (95% CI, 0.47–1.35) and the uHR in tertile 3 was 1.36 (95% CI, 0.85–2.18) (*P*_trend_=0.09) (Table 5).

**Table 5:** HRs for All‐Cause Mortality by Tertiles of VAT

Variable	Median (Minimum–Maximum) VAT, g	No. of Participants	No. of Events	Unadjusted HR (95% CI)
VAT				
Tertile 1	576 (5–925)	1098	30	1.0 (Referent)
Tertile 2	1318 (976–1762)	1098	23	0.80 (0.47–1.35)
Tertile 3	2362 (1764–7033)	1098	42	1.36 (0.85–2.18)
*P* value for trend				0.09

HR indicates hazard ratio; and VAT, visceral adipose tissue.

## DISCUSSION

In this prospective study, greater VAT, but not lower muscle density, was independently associated with increased risk of stroke and MI in community‐dwelling 70‐year‐old men, although there were no associations with all‐cause mortality. These findings indicate that despite being a small fat depot in absolute measures, VAT is a strong obesity‐related predictor of cardiovascular risk in 70‐year‐old men, and by implication, that strategies for decreasing VAT may play a role in prevention of incident CVD.

We found that greater VAT was associated with around 60% increased risk of stroke or MI in the total cohort after adjustment for sex, lifestyle factors, socioeconomic status, cardiovascular risk factors, CVD history, medications, total fat mass, and muscle density. The result was confirmed when excluding early cases and participants with short follow‐up and CVD history and is similar to that of a small prospective study in which greater VAT was associated with 60% increased risk of coronary artery disease in 60‐year‐old men.^39^ Our risk estimate is also higher than the one observed in the Health, Aging and Body Composition (ABC) Study, including 2503 men and women aged 70 to 79 years, in which greater VAT was associated with 20% increased risk of incident MI.^18^ Interestingly, we observed a significant VAT‐sex interaction, where greater VAT conferred a nearly 2‐fold increased risk of stroke or MI in older men, whereas there was no association in women. Previously, the cross‐sectional association of obesity with CVD, hypertension, and dyslipidemia was shown to be attenuated with higher age, especially in women.^40^ In addition, in the Framingham Heart Study, which comprised participants across a wide age range but with a similar mean VAT mass as in our population,^41^ the association of VAT with incident CVD appeared stronger in men than in women. In contrast, in the Health ABC study, the association between VAT and MI was significant in older women, but not men.^18^ However, older men have around twice the amount of VAT compared with women,^42^ which was the case also in the present study, yet, in the Health ABC Study there were no sex differences in VAT among those who experienced an MI. Thus, the lack of association in older men in that study could be attributable to selection bias, where men with greater VAT had already experienced earlier CVD mortality. Although an additional study found no association between VAT and incident CVD in older men,^19^ those participants were much older already at baseline (76 years) as well as followed up for a mean of 8 years. This is important to note given that the association between obesity and morbidity becomes weaker in the very old.^40^ It is also possible that individuals who have lived until a high age may carry VAT that is less detrimental, as not only the quantity, but also the quality, of VAT influences cardiovascular risk.^43^ With this in mind, our result suggests that the association of VAT with CVD in older people appears strong, especially in the younger of older individuals, and in men, and weakens with increasing age. Yet, to draw definitive conclusion on the age and sex interactions, studies including even larger study samples with a wide age range are needed.

Our findings for VAT have clinical implications given that the age‐associated shift toward increased VAT is exacerbated by behavioral factors, such as decreased physical activity.^13^ This would suggest that lifestyle interventions targeting VAT in older people may have implications for CVD prevention. For example, meta‐analyses show that exercise interventions can effectively decrease VAT by around 6%, and that effects can be attained independent of weight loss.^44^, ^45^ In a recent randomized study including 70‐year‐old individuals with central obesity,^46^ we found a similar effect size with simultaneous gains in muscle mass in the male participants,^46^ which becomes of particular interest given the sex‐specific findings of the present study. Thus, exercise may be a feasible strategy to decrease VAT in 70‐year‐old men while preserving vital muscle mass, which, based on the results of the present study, may have a favorable impact on their risk of CVD.

There are several plausible mechanisms that could explain the link between VAT and atherosclerotic CVD. VAT has a high lipolytic activity, leading to an increase in circulating free fatty acids, which promotes endothelial dysfunction.^47^, ^48^ Because of its anatomical position, VAT is drained through the portal vein, leading to increased free fatty acids and proinflammatory cytokines in the liver, which may promote insulin resistance and increased secretion of very‐low‐density lipoproteins.^47^, ^48^ Furthermore, VAT is characterized by high infiltration of macrophages, which increases the production and secretion of reactive oxygen species and proinflammatory adipokines, such as interleukin‐6 and tumor necrosis factor‐α,^47^, ^48^ which are associated with chronic inflammation, insulin resistance, and endothelial dysfunction.^47^, ^48^ Interleukin‐6 and tumor necrosis factor‐α also inhibit the production of adiponectin, which in contrast to interleukin‐6 and tumor necrosis factor‐α is inversely associated with VAT and has antiatherogenic, anti‐inflammatory, and insulin‐sensitizing properties. Moreover, VAT is associated with increased plasma levels of plasminogen activator inhibitor‐1, which contributes to thrombosis in the atherosclerotic process.^47^, ^48^

In contrast, we found no significant association between muscle density and incident stroke or MI after multivariable adjustment in our main analysis. In a longitudinal study, lower muscle density was associated incident hypertension in middle‐aged and older individuals.^49^ However, these individuals were exclusively of African ancestry, which have greater skeletal muscle fat infiltration compared with White individuals,^49^ thus potentially contributing to the lack of association in the present population. Another difference is that our study included adjustment for several other risk factors, such as blood pressure, lipids, prescription medications, and objectively measured physical activity. Because our results showed a significant association before adjustment for these factors, it is possible that the association in the previous study would have been attenuated with adjustment for these factors. Another possibility is that assessment of muscle density simply has little additive value in terms of cardiovascular risk prediction beyond that of VAT. This was the case in our main analysis, and previous cross‐sectional studies have shown that the association of lower muscle density with insulin resistance, metabolic risk factors, and coronary artery calcification may largely be explained by the joint presence of greater VAT.^15^, ^21^, ^22^ Despite all this, we cannot rule out the possibility that muscle density is a risk factor for CVD, as there was a significant association in our sensitivity analysis. Because our study was, to our knowledge, the first to investigate muscle density and incident stroke or MI in older adults, similar studies with even longer follow‐up and larger study samples are warranted.

In terms of all‐cause mortality and VAT in older adults, results from previous studies are conflicting.^26^, ^28^, ^29^ In the present study, greater VAT was not associated with higher all‐cause mortality, which supports the findings from a longitudinal study.^27^ However, that study may potentially also be influenced by selection bias as those participants were on average 78 years when the follow‐up, which lasted up to 12 years, started.^27^ In support of this argument, studies on older adults of younger age have found other results. In a study of >130 000 older adults aged 60 to 69 years, anthropometric indexes of central obesity were associated with higher mortality,^50^ and a meta‐analysis of 58 000 older adults aged 65 to 74 years found similar results, and a J‐shaped association.^51^ This is of interest as our findings also suggested a kind of J‐shaped dose‐response association between greater VAT and higher all‐cause mortality, although the association was not statistically significant and should be cautiously interpreted as the analyses were unadjusted. Nevertheless, even though VAT was not a risk factor for mortality, it was associated with increased risk of stroke or MI, which, in turn, may increase the risk of death; hence, greater VAT does appear to exert harmful effects. To further investigate the dose‐response trajectory of VAT and mortality in detail and establish whether VAT is a definitive risk factor for mortality in older adults, studies including even larger samples as well as pooling of their results would be valuable.

We did not find an association between midcalf muscle density and higher all‐cause mortality, similar to a previous study.^25^ Two other studies found an association between midcalf muscle density and mortality, but these included either much older men than ours or older men of African ancestry,^23^, ^24^ which may explain the discrepancy in results. Yet, larger studies including both older men and women with different ethnicities have observed an association between thigh, paraspinous, and abdominal muscle density and mortality.^52^, ^53^, ^54^ Whether muscle density in certain muscle groups exerts stronger associations is not clear and warrants further investigation.

This study has several strengths, including the assessment of VAT and muscle density in nearly 3300 individuals and the prospective follow‐up for study end points using national registers. DXA‐measured VAT and peripheral quantitative computed tomography–measured muscle density are considered good alternatives to computed tomography and magnetic resonance imaging, with certain beneficial characteristics, including their short scan time, as well as low cost and radiation.^55^, ^56^ Next, because the NPR includes all diagnoses in inpatient and secondary outpatient care in Sweden, and all cases of death are recorded in the Cause of Death Register, we had zero loss to follow‐up, minimizing the risk of selection bias. In addition, the precision of diagnoses of stroke and MI in the NPR is high, which means that the risk of information bias attributable to measurement error was minimized. Moreover, although unmeasured or residual confounding may always be present in an observational study, we were able to adjust our statistical models for a wide number of potential confounders, which were obtained from the registers as well as through clinical examinations in HAI. Together, these factors increase the internal validity of our findings. Another strength is the large population‐based sample and the high participation rate, meaning that the results could potentially be generalized to other cohorts of community‐dwelling 70‐year‐old individuals. This increases the external validity of the findings. There are also some limitations, with the most obvious one being the observational design, meaning that causality cannot be fully established. Although we adjusted for several covariates, we lacked information on other variables of interest, such as inflammatory markers and other types of fat, such as fat in the heart and liver. Furthermore, although the aim of this study was to study the impact of VAT, we are aware that also other factors related to ageing and body composition are important and may contribute, particularly reduced muscle strength,^57^ which has been associated with CVD and mortality.^58^, ^59^ Next, muscle density was measured in the midcalf, and it is possible that muscle density in other muscle groups may present different associations. Given the paucity of data in this field, it would be valuable if future studies could perform comparative investigations of muscle density in different muscle groups. Although statistical power was unlikely to have been a major issue in the present study, an even larger number of cases would have allowed for a more elaborated investigation of the shape of the dose‐response trajectories. Finally, average follow‐up was relatively short and longer follow‐up may potentially have led to different results, although the results were generally consistent when follow‐up time was restricted to at least 1 year.

## Conclusions

This prospective study shows that greater VAT is associated with increased risk of stroke or MI in community‐dwelling 70‐year‐old men, independent of a wide number of potential confounders including total fat mass and skeletal muscle fat infiltration. With the limitation of being an observational study, these findings suggest that preventive measures to decrease VAT in 70‐year‐old men may potentially lower their risk of incident CVD.

## Sources of Funding

This work was supported by the Swedish Research Council (grant 2016‐02589 to Dr P. Nordström).

## Disclosures

None.
